# Adhesion, biofilm formation, cell surface hydrophobicity, and antifungal planktonic susceptibility: relationship among *Candida* spp.

**DOI:** 10.3389/fmicb.2015.00205

**Published:** 2015-03-12

**Authors:** Ana Silva-Dias, Isabel M. Miranda, Joana Branco, Matilde Monteiro-Soares, Cidália Pina-Vaz, Acácio G. Rodrigues

**Affiliations:** ^1^Department of Microbiology, Faculty of Medicine, University of PortoPorto, Portugal; ^2^Cardiovascular Research and Development Unit, Faculty of Medicine, University of PortoPorto, Portugal; ^3^CINTESIS – Center for Health Technology and Services Research, Faculty of Medicine, University of PortoPorto, Portugal; ^4^CIDES, Department of Information and Decision Sciences in Health, Faculty of Medicine, University of PortoPorto, Portugal; ^5^Department of Microbiology, Centro Hospitalar S. JoãoPorto, Portugal; ^6^Burn Unit and Department of Plastic and Reconstructive Surgery, Centro Hospitalar S. JoãoPorto, Portugal

**Keywords:** *Candida*, adhesion, biofilm, non-*albicans* species, antifungal susceptibility, cell surface hydrophobicity, flow cytometry

## Abstract

We have performed the characterization of the adhesion profile, biofilm formation, cell surface hydrophobicity (CSH) and antifungal susceptibility of 184 *Candida* clinical isolates obtained from different human reservoirs. Adhesion was quantified using a flow cytometric assay and biofilm formation was evaluated using two methodologies: XTT and crystal violet assay. CSH was quantified with the microbial adhesion to hydrocarbons test while planktonic susceptibility was assessed accordingly the CLSI protocol for yeast M27-A3 S4. Yeast cells of non-*albicans* species exhibit increased ability to adhere and form biofilm. However, the correlation between adhesion and biofilm formation varied according to species and also with the methodology used for biofilm assessment. No association was found between strain's site of isolation or planktonic antifungal susceptibility and adhesion or biofilm formation. Finally CSH seemed to be a good predictor for biofilm formation but not for adhesion. Despite the marked variability registered intra and inter species, *C. tropicalis* and *C. parapsilosis* were the species exhibiting high adhesion profile. *C. tropicalis*, *C. guilliermondii*, and *C. krusei* revealed higher biofilm formation values in terms of biomass. *C. parapsilosis* was the species with lower biofilm metabolic activity.

## Introduction

Invasive candidiasis is the third to fourth most frequent health care related infection (HCRI) in hospitals worldwide. *C. albicans* agent accounts for more than 50% of mucocutaneous and systemic yeast infections (Pfaller and Diekema, 2007; Lai et al., 2008; Pfaller, 2012). Nevertheless, non-*albicans* species prevalence is increasingly becoming more relevant. Recent advances in the management and control of these infections have been achieved, namely with new antifungal therapeutics. Still, HCRIs caused by yeasts remains extremely high and with a poor outcome, since associated mortality rate ranges from 30 to 50% (Viudes et al., 2002; Pfaller and Diekema, 2007; Pfaller, 2012). A common problem involving the treatment of *Candida* infections is therapeutic failure, particularly due to clinical resistance to antifungals. *Candida* species are known to develop several mechanisms, initially to tolerate and ultimately to confer antifungal resistance. These mechanisms are described and well characterized in the case of free floating planktonic cells. However, resilient infections are invariably associated with two important virulence factors: adhesion and biofilm formation (Ramage et al., 2001; Kuhn et al., 2002b; Uppuluri et al., 2009).

The ability to gain access to deep tissues either in healthy and immunocompromised humans is likely to result in promoted adhesion to host tissues or to medical indwelling devices such as cardiovascular catheters, endotracheal tubes and cerebrospinal-fluid shunts. This assumption is supported by the high correlation found between central venous catheterization and haematogenous infections caused by *Candida* (Kojic and Darouiche, 2004; Ramage et al., 2006; Uppuluri et al., 2009).

Microbial adhesion is considered the first step for biofilm formation. This structure constitutes a protective milieu against environmental stresses and human host defenses. It is documented that approximately 65% of all clinical infections are associated with microbial biofilm formation on the surface of tissues, organs or medical devices (Kojic and Darouiche, 2004; Uppuluri et al., 2009; Sousa et al., 2011). Most importantly biofilm-associated microorganism's exhibit dramatically decreased susceptibility to antimicrobial agents. This fact triggers serious clinical concerns, not only in the treatment of patient infection but also for public health (Kuhn et al., 2002b; Uppuluri et al., 2009, 2010; Ramage et al., 2012).

Considerable knowledge is already available regarding *C. albicans* adhesion and biofilm formation; nevertheless it's scarce which concerns to other *Candida* species. The aim of this study was to characterize a large number of clinical isolates belonging to the most clinical relevant *Candida* species, regarding adhesion performance, biofilm formation ability, cell surface hydrophobicity and antifungal susceptibility profile.

## Materials and methods

### Strains

Forty nine *C. albicans*, 48 *C. glabrata*, 47 *C. parapsilosis*, 24 *C. tropicalis*, 8 *C. krusei*, and 8 *C. guilliermondii* isolated from several body sites were used (Figure S1). *C. albicans* type strain ATCC 90028 was also used as a control. The clinical *Candida* isolates were obtained from patients from Centro Hospitalar São João, Porto. All strains were identified using the VITEK 2 system (bioMérieux, Vercieux, France) and kept frozen in Yeast Peptone Dextrose medium (Formedium, Hunstanton, England) (YPD) supplemented with 40% glycerol at −70°C until testing. Prior to each experiment, the microorganisms were sub-cultured twice on Sabouraud agar (Liofilchem, Italy), 35°C, 24 h, to assess the purity of the culture and its viability.

### Adhesion assay

*Candida* strains were grown overnight in Sabouraud broth at 37°C and 180 rpm. Cells were harvested by centrifugation (10,000 g, 5 min), washed twice with phosphate buffer saline (PBS) (Sigma-Aldrich) and standardized to 2 × 10^6^ cells/ml in PBS.

Adhesion was evaluated by means of a flow cytometric assay, as previously described (Silva-Dias et al., 2012). Briefly, yeast cells suspensions, at the above mentioned concentration were mixed with 2 × 10^8^ microspheres/ml of uncoated carboxylated highly green fluorescent polystyrene microspheres (Molecular Probes) and incubated at room temperature for 30 min, with agitation (150 rpm). Following incubation, yeast cell suspensions were vortexed and 50,000 events were analyzed in a FACSCalibur flow cytometer (FACSCalibur BD Biosciences, Sydney). Results were expressed using two parameters: (a) percentage of cells with microspheres attached and (b) distribution pattern.

Strain's adhesion results are a representative of at least three independent experiments, performed in triplicate.

### Biofilm assay

*Candida* strains were grown overnight in Sabouraud broth at 37°C and 180 rpm. Cells were harvested by centrifugation (10,000 g, 5 min), washed with PBS and standardized to 1 × 10^6^ yeast cells/ml in RPMI-1640 medium supplemented with L-glutamine and buffered with MOPS acid (Sigma-Aldrich). Following, 1 ml aliquots of this yeast cell suspension were placed in the wells of a 12-well polystyrene microplates and incubated for 24 and 48 h at 37°C (Pierce et al., 2008). After incubation biofilm in each well was quantified by two distinct methodologies: the semi quantitative 2,3-bis-(2-methoxy-4-nitro-5-sulfophenyl)-2H-tetrazolium-5-carboxanilide (XTT) reduction assay and the crystal violet (CV) assay, accordingly to previously described protocols (Peeters et al., 2008; Pierce et al., 2008).

Strain's biofilms results are a representative of at least three independent experiments, performed in triplicate.

### Cell surface hydrophobicity assay

Cell surface hydrophobicity (CSH) was assessed using the microbial adhesion assay to hydrocarbons (MATH) (Rosenberg, 1984). Briefly, yeast cells grown overnight at 37°C, were harvested and washed twice with PBS. A yeast cell suspension displaying an OD_600 nm_ between 0.4 and 0.5 was prepared in PBS (A_0_); 3 ml of this yeast suspension was overlaid by 0.4 ml of the hydrophobic hydrocarbon, n-hexadecane (Sigma-Aldrich). After vigorous vortexing, phases were allowed to separate for 10 min at 30°C and the OD_600 nm_ of the aqueous phase was measured (A_1_). The percentage of hydrophobicity was calculated as follows: hydrophobicity (%) = [1−(A_1_/A_0_)] × 100. All assays are a representative of at least three independent experiments, performed in triplicate.

### Antifungal susceptibility profile

Antifungal susceptibility testing was performed for three antifungals: fluconazole (FLC), amphotericin B (AMB) and caspofungin (CAS). The minimal inhibitory concentration (MIC) for each drug was determined according to the CLSI (Clinical Laboratory Standards Institute) reference protocol M27-A3 S4 for yeasts. The susceptibility breakpoints for FLC and CAS were those established by CLSI. Since there is no standard breakpoints for AMB according to the literature we considerate S ≤ 1 μg/ml and R for >1 μg/ml (Pfaller et al., 2003).

### Statistical analysis

Statistical analysis started with distribution normality assessment by histogram evaluation. Since some variables' distribution was not asymmetric, both parametric and non-parametric tests were used. According to the variable distribution, Student's *t* test or the Wilcoxon Signed Ranks Test was used for the comparison between 24 and 48 h biofilm production and between species' biofilm measures at 24 and 48 h. For the comparison of the adhesion profile (low, intermediate or high) between species the X^2^ test was used. In the same way, correlation coefficients (r) were calculated using the Pearson Correlation or Spearman's Rank Correlation. A two-tailed analysis was conducted and a *p*-value inferior to 0.05 was considered as statistically significant.

All statistical analysis was performed using the SPSS software (v. 20.0).

## Results

### Characterization of *Candida* spp. adhesion profile

**Figure 1:**
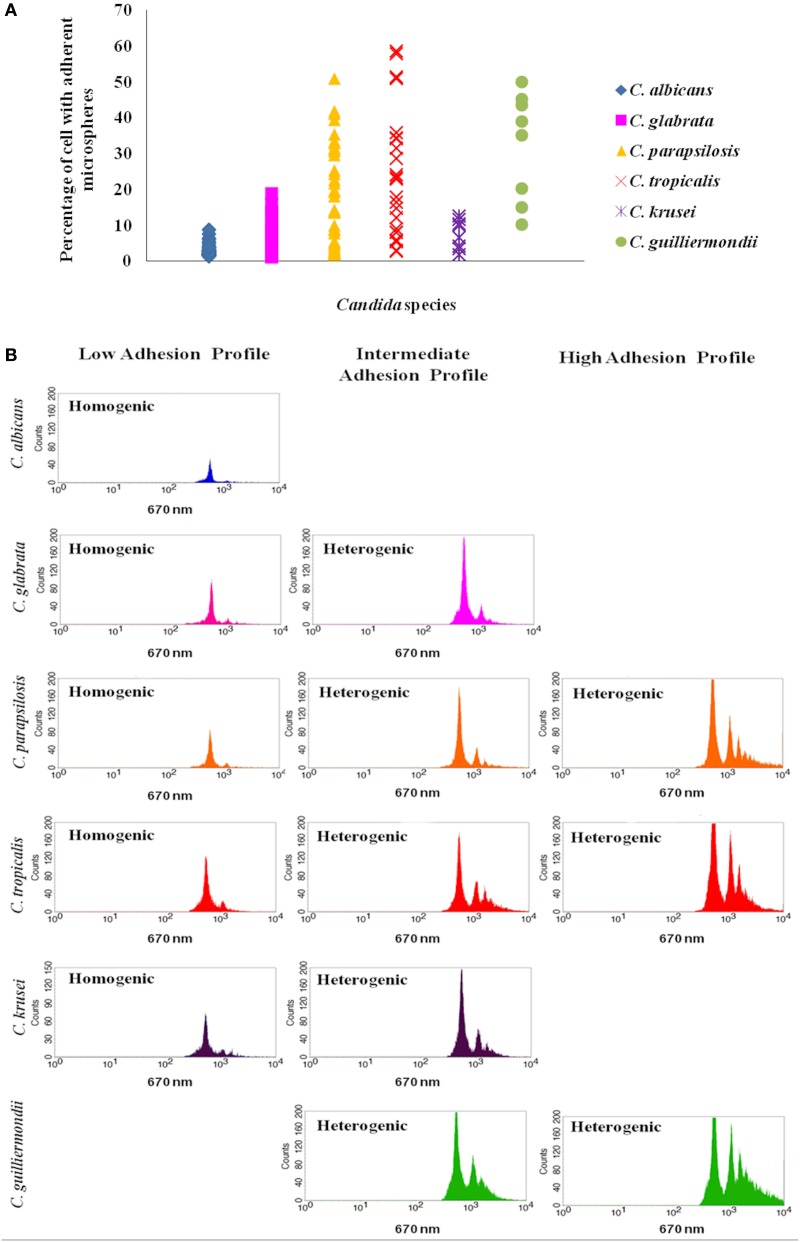
**Representation of *Candida* adhesion profiles. (A)** The species with higher percentage of cells with adherent microspheres are *C. guilliermondii*, *C. tropicalis* and *C. parapsilosis*. Results represent the mean of at least 3 independent experiments, performed in triplicate. **(B)** Representative histograms illustrate the different distribution patterns that characterize the low, intermediate and high adhesion profiles displayed by each species: homogenic (a homogenous distribution pattern characterizes a population of yeast cells bound to the same number of microspheres, frequently binding to a single microsphere) and heterogenic (a heterogeneous pattern displays the presence of different peaks beyond the third logarithmic decade and indicates that more than a single microsphere is attached to each yeast cell) distribution patterns.

The distinct *Candida* species displayed a variable adhesion profile to polystyrene microspheres. Regarding *C. albicans*, the percentage of cells with microspheres ranged from 1.42 to 8.92%, with a mean value of 2.90% (±1.62). All *C. albicans* strains exhibit a homogeneous adhesion pattern, meaning that each yeast cell was bound to a single microsphere (Figure 1, Table S1). Among *C. glabrata* tested strains, a higher variability was found; in 91.7% of the cases the percentage of cells with adherent microspheres ranged from 1.1 to 9.95%. Nevertheless, 4 strains exhibited a heterogeneous adhesion pattern, with higher percentages of adherent cells (Figure 1, Table S1). *C. parapsilosis* was found to be the most heterogeneous species concerning adhesion; the percentage of cells with adherent microspheres was highly variable, ranging from 1.78 to 51.05% (Figure 1, Table S1). Similar to *C. parapsilosis, C. tropicalis* strains also presented both a homogenous and heterogeneous patterns, but with higher adhesion values, ranging from 2.94 to 58.70% (Figure 1, Table S1). Regarding *C. krusei* and *C. guilliermondii* fewer isolates were tested; nevertheless *C. krusei* consistently displayed a homogenous pattern with percentages of adhesion ranging from 1.93 to 12.57%, while *C. guilliermondii* isolates mainly fit in the heterogeneous pattern with higher percentages of adherent cells ranging from 15.13 to 50.13% (Figure 1, Table S1).

The variability between the distinct *Candida* isolates led to the creation of a new category of adhesion profile in order to classify the strains that did not fit in the low or in the high adhesion profile. Therefore, taking into account the values found, three adhesion profiles were established: the low, the intermediate and the high adhesion profiles (Table S2). Based in these categories, strains were classified and the results are summarized in Table 1. All *C. albicans* strains presented a low adhesion profile. Among *C. glabrata* strains tested, 91.7% showed a low adhesion profile, while 8.2% displayed an intermediate profile. *C. parapsilosis* and *C. tropicalis* were the most heterogeneous strains; 51.1% of *C. parapsilosis* isolates displayed low profile, 23.4% intermediate profile and 25.5% a high adhesion profile. *C. tropicalis* adhesion profiles were distributed as follows: 33.3% low, 29.2% intermediate and 37.5% high adhesion profiles (Table 1 and Table S1).

**Table 1:** ***Candida* species adhesion profile**.

**Adhesion profile Species**	**Total (*n*)**	**Low [*n* (%)]**	**Intermediate [*n* (%)]**	**High [*n* (%)]**
*Candida albicans*	50	50 (100.0)	0 (0.0)	0 (0.0)
*Candida glabrata*	48	44 (91.7)	4 (8.2)	0 (0.0)
*Candida parapsilosis*	47	14 (51.1)	11 (23.4)	12 (25.5)
*Candida tropicalis*	24	8 (33.3)	7 (29.2)	9 (37.5)
*Candida krusei*	8	4 (50.0)	4 (50.0)	0 (0.0)
*Candida guilliermondii*	8	3 (37.5)	5 (62.5)	0 (0.0)

For each Candida strain the percentage of cells with adherent polystyrene microspheres was quantified and the homogenic or heterogenic adhesion pattern was attributed. Based in these attributes an adhesion profile was established. In this table the adhesion profiles displayed by each species were summarized in order to compare the species adhesion tendency.

Considering the tested clinical species, *C. tropicalis* and *C. parapsilosis* were the only species that displayed high adhesion profile. *C. albicans* consistently exhibited the low adhesion profile and no significant differences were found for the other tested species (Figure 1, Table 1).

In order to understand whether there was a relation between yeast adhesion ability and the site of isolation, strains of each species were distributed according to the respective isolation site and the possible association between these two variables was assessed. Generally, no association was found between a higher adhesion profile and the site of isolation. Interestingly, only for *C. parapsilosis* an association was found: strains collected from mucocutaneous sites invariably displayed a high adhesion profile.

### Biofilm formation ability

*Candida* biofilms were quantified at two different time points, 24 and 48 h, with two different methodologies: CV assay, which measures the total biomass of the biofilm and the XTT assay, which measures the biofilm metabolic activity.

Regarding biofilm biomass at 24 h *C. parapsilosis* produced lower amount of biomass than *C. tropicalis* and *C. guilliermondii*; *C. albicans* and *C. glabrata* produced lower biomass than *C. tropicalis*, *C. krusei*, and *C. guilliermondii*. *C. tropicalis* produced higher amount of biomass than *C. krusei*.

Relating to the 48 h time point *C. parapsilosis* showed lower biomass production than *C. tropicalis*; once again *C. albicans* and *C. glabrata* produced lower quantity of biomass than *C. tropicalis*, *C. krusei* and *C. guilliermondii*. *C. tropicalis* showed higher total biomass than *C. guilliermondii*.

**Figure 2:**
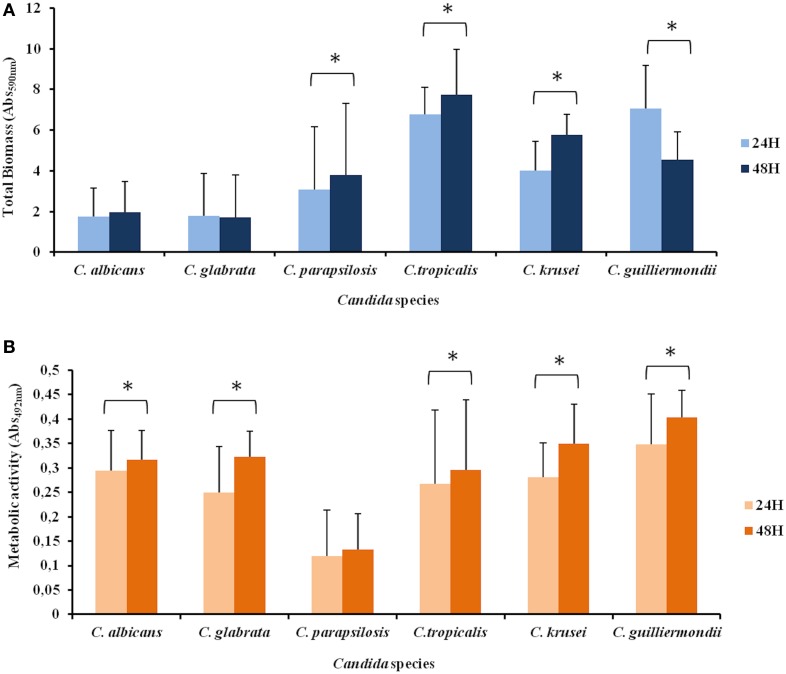
**Biofilm formation by different *Candida* species**. Biofilm was quantified colorimetrically by two different methodologies: **(A)** Crystal violet assay, that measures biofilm total biomass and **(B)** XTT assay, which measures biofilm metabolic activity. Error bars represent the standard deviation among results for different isolates. Each isolate was tested for its ability to form biofilm at least 3 times. Asterisks represent cases were a statistically significant difference in the values at 24 and 48 h were observed. Due to asymmetric distribution and sample size, in several comparisons non-parametric tests were used.

Considering the two studied time points, no differences were found for biomass production among *C. albicans* and *C. glabrata*; *C. parapsilosis*, *C. tropicalis*, and *C. krusei* presented more biomass formation at 48 h while *C. guilliermondii* decreased the total biomass from 24 to 48 h (*p* < 0.05) (Figure 2A).

Concerning biofilm metabolic activity, *C. parapsilosis* showed lower values than all species at 24 and 48 h time points. No differences were found among the other species at 24 h. At 48 h, *C. albicans* showed higher metabolic activity than *C. glabrata* and there were no differences among the other species. High intraspecies variability was found.

All species showed a higher metabolic activity at 48 h (*p* < 0.05), except for *C. parapsilosis* that no difference was found between the two time points (Figure 2B).

No correlation was found between the two methodologies used for biofilm quantification.

The association between 48 h biofilm formation and the site of isolation was investigated for both methodologies; nevertheless no significant association was found for any species.

Since adhesion is determinant for biofilm formation, the correlation between the percentage of cells with adherent microspheres and biofilm formation at 24 and 48 h was evaluated. A significant correlation was found between adhesion and biofilm biomass for *C. glabrata* (r^2^ 0.027) and *C. parapsilosis* (r^2^ 0.602) at 24 h and for *C. glabrata* (r^2^ 0.016), *C. parapsilosis* (r^2^ 0.608), and *C. tropicalis* (r^2^ 0.097) at 48 h time point. Correlation between adhesion and biofilm metabolic activity was found only for *C. albicans* (r^2^ 0.169 at 24 h and r^2^ 0.172 at 48 h) at both time points. This comparison was not performed for *C. krusei* and *C. guilliermondii* due to the reduced number of isolates tested.

### *Candida* hydrophobicity and its relation with adhesion and biofilm formation

**Figure 3:**
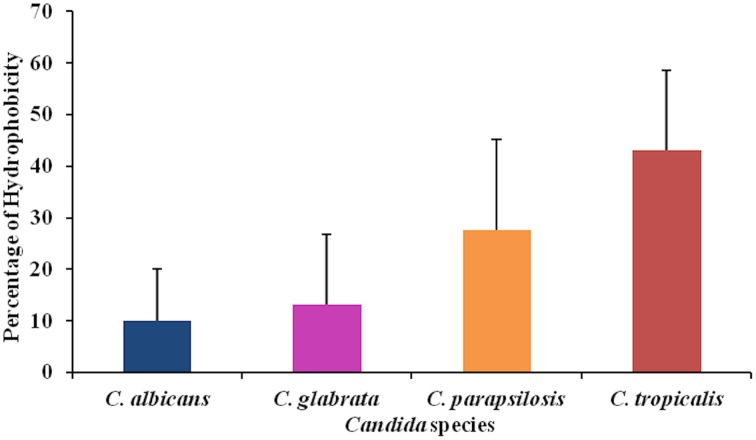
***Candida* hydrophobicity was measured according MATH test**. Results are representative of the mean results of 15 strains for each species. Each strain was tested three times in different occasions.

Together with adhesion ability, hydrophobicity is another characteristic usually related with biofilm formation. Thus, hydrophobicity of yeast cells was measured for the most clinical relevant *Candida* species: *C. albicans*, *C. glabrata*, *C. parapsilosis*, and *C. tropicalis*. *C. tropicalis* was the species which displayed the higher values of hydrophobicity. Once more high intraspecies variability was found (Figure 3).

Correlation between adhesion and hydrophobicity was found only for *C. parapsilosis* (r^2^ 0.331). A positive correlation was verified between hydrophobicity and biofilm biomass for *C. parapsilosis* (r^2^ 0.384) and *C. glabrata* (r^2^ 0.623); for *C. albicans* (r^2^ 0.246), *C. parapsilosis* (r^2^ 0.250) and *C. tropicalis* (r^2^ 0.341) hydrophobicity was positively correlated with biofilm metabolic activity.

### Antifungal susceptibility of planktonic cells

*Candida* planktonic cells were tested regarding its susceptibility to FLC, AMB, and CAS. Taking into account the established breakpoints, 6 *C. albicans* were resistant to FLC and 1 was SDD; 3 strains were resistant to CAS and 1 intermediate. No resistance to AMB was found. Regarding *C. glabrata*, 1 strain was resistant to FLC and 47 were SDD; 1 strain was resistant to AMB; 8 strains were resistant to CAS and 7 were intermediate. Two strains of *C. parapsilosis* were resistant to FLC and one was SDD; 1 was resistant to AMB; 4 were resistant to CAS and 14 were intermediate. In the case of *C. tropicalis*, 6 strains were resistant to FLC and one was SDD; 4 were resistant to CAS and 4 were intermediate; all strains were susceptible to AMB. All *C. krusei* strains were resistant to FLC, 1 strain was resistant to CAS and 5 were intermediate. Six *C. guilliermondii* strains were resistant to CAS and 1 was intermediate (Table S3).

No association was found between antifungal resistance of planktonic cells and higher adhesion profile or biofilm formation, for any species.

## Discussion

Invasive or mucocutaneous candidosis is commonly caused by *C. albicans*. Due to its clinical prevalence this species is the best characterized among the genus *Candida*. Non-*albicans* species have been increasingly identified as infection agents; however its respective virulence attributes are poorly described (Estivill et al., 2011; Silva et al., 2011, 2012).

Thus, an extensive characterization of important virulence factors, like adhesion ability, biofilm formation, hydrophobicity and antifungal susceptibility was carried out. This study comprises 184 clinical isolates belonging to different *Candida* species, namely *C. albicans*, *C. glabrata*, *C. parapsilosis*, *C. tropicalis*, *C. krusei*, and *C. guilliermondii*. Adhesion is considered an important virulence attribute once it represents the first step for persistent colonization, biofilm formation and establishment of disease. We verified that, despite being the species most often related with fungal infection, *C. albicans* was the less adherent species, followed in increasing values by the non-*albicans* species, particularly *C. tropicalis* and *C. parapsilosis*. Comparatively to *C. albicans*, non-*albicans* strains were already characterized as displaying higher adhesion ability (Rotrosen et al., 1986; Luo and Samaranayake, 2002; Negri et al., 2010; Silva et al., 2010b). Among these, considerable intraspecies variation was found between adhesion profiles. Higher or lower adhesion profile of one species compared with other is extremely dependent of the substrate and growth conditions used (Ten Cate et al., 2009; Negri et al., 2010; Silva et al., 2010a,b; Cuellar-Cruz et al., 2012). The different appetencies to adhere could also be attributed to species and strain distinct cell wall composition.

*C. albicans* revealed the lower adherence values, although we should emphasize that adhesion was assessed mainly in the yeast form while other forms of growth might display higher adhesion profiles, namely hyphae, pseudohyphae, and opaque cells. It has been described that *C. albicans* is more adherent in the hyphae form, when expressing adhesins like Als1, Als3, and Hwp1 (Nobile and Mitchell, 2006; Dranginis et al., 2007; Tronchin et al., 2008).

Another factor related to the cell wall composition is CSH, which is usually considered a good indicator of adhesion ability. Some previous studies described a positive correlation between hydrophobicity and adhesion to plastic and host cells while other reports couldn't find such relation (Panagoda et al., 2001; Gallardo-Moreno et al., 2002; Luo and Samaranayake, 2002; Samaranayake et al., 2003; Blanco et al., 2010; Raut et al., 2010; Yoshijima et al., 2010). We only found correlation between adhesion and hydrophobicity for *C. parapsilosis* strains. Such result is in accordance with other study where hydrophobicity was associated to the initial adhesion of this species to acrylic surfaces (Panagoda et al., 2001). CSH was very variable among the distinct isolates of each species, but in general the most hydrophobic species were *C. tropicalis*, followed by *C. parapsilosis*, *C. glabrata*, and *C. albicans*. Different rankings of hydrophobicity between these species were already proposed. But we should have in mind that different quantification methodologies, different growth conditions, or different temperatures, may certainly contribute for a different ranking achievement. Thus, despite CSH may affect virulence in several ways, we concluded that CSH alone was not a predictor of adhesion to polystyrene.

This large survey regarding *Candida* adhesion forced the appearance of a new category in the adhesion profile score previously described. In fact, some strains didn't fit in the low/high adhesion profile score and therefore an intermediate class was created. The implementation of these new breakpoints allowed us to easily classify and characterize a strain in a more informative way comparatively to other methodologies.

Biofilm formation ability, an important attribute of virulence, was also quantified. In addition, we have consistently characterized adhesion and biofilm formation ability of *C. krusei* and *C. guilliermondii*, for the first time. Despite their lower frequency of isolation *C. guilliermondii* is an important agent of mucocutaneous infection and *C. krusei* infections are associated with high mortality rates. Our results showed that *C. parapsilosis* displayed the lower values of metabolic activity. Regarding biofilm biomass, *C. tropicalis, C. guilliermondii*, and *C. krusei* were the species with higher biomass production followed by the other tested species: *C. parapsilosis*, *C. glabrata*, and *C. albicans*. The species biomass ranking we propose agrees with previous studies that nominate non-*albicans* strains as biofilms producers with higher biomass and distinct extracellular matrix (Al-Fattani and Douglas, 2006; Parahitiyawa et al., 2006; Estivill et al., 2011; Melo et al., 2011; Silva et al., 2011). No difference was found in biomass production between the two time points studied in the case of *C. albicans* and *C. glabrata.* This result suggests that these species are faster biofilm formers, indicating that biofilm becomes completely established in the first 24 h. *C. parapsilosis*, *C. tropicalis* and *C. krusei* exhibited more biomass at 48 h, suggesting that these species are slower biofilm formers. Curiously, in the case of *C. guilliermondii*, the biomass decreases from 24 to 48 h. Despite the use of a static assay, this might be due to biofilm dispersal or detachment of cells for other places colonization.

It is intuitive to infer that cellular metabolic activity should correlate with biomass, and in fact some studies found this correlation (Jin et al., 2003; Li et al., 2003; Melo et al., 2011). Nevertheless, in our study, we couldn't find a correlation between XTT and CV assays at 24 or 48 h. Other authors found the same lack of correlation, suggesting that this finding can occur for two reasons. First, biofilm is composed of several cell layers and the basal ones may not be as active as the ones on the top of the biofilm. Second, the inherent metabolic activity of each strain (Henriques et al., 2006; Silva et al., 2010a), the rate of metabolism of XTT may vary from species to species. It has been shown that the XTT metabolism rate displayed by *C. parapsilosis* is slower when compared to *C. albicans* (Kuhn et al., 2002a; Parahitiyawa et al., 2006; Silva et al., 2010a). Thus, XTT seems to be a good quantification method for comparisons within the same strain, while CV can be used for comparison inter and intraspecies.

In previous reports, biofilm formation has been associated with CSH (Li et al., 2003; Blanco et al., 2010). In our study, a positive correlation was found between biofilm biomass and CSH in the case of *C. glabrata* and *C. parapsilosis.* Interestingly, correlation between metabolic activity and CSH was only found for *C. albicans* and *C. tropicalis*.

While some studies claim that some isolates are more prone to adhere and form biofilm depending on their isolation site, the vast majority didn't found any association (Shin et al., 2002; Hasan et al., 2009; Silva et al., 2010a, 2011; Mohandas and Ballal, 2011). Only in *C. parapsilosis* a positive association between high adhesion profile and its mucocutaneous provenance was found. No association was found regarding adhesion, biofilm formation and planktonic antifungal susceptibility profiles, discouraging the idea that planktonic antifungal resistance influences adhesion and biofilm formation abilities.

Our large survey of *Candida* clinical isolates assessing adhesion, CSH, biofilm formation and antifungal susceptibility profile, allowed the characterization of each isolate, giving good indications of the species specific tendency. For the first time a comprehensive study regarding adhesion to polystyrene was performed with *C. krusei* and *C. guilliermondii* clinical isolates, adding knowledge regarding this species virulence attributes.

### Conflict of interest statement

The authors declare that the research was conducted in the absence of any commercial or financial relationships that could be construed as a potential conflict of interest.
