# Supplementary material for: Adhesion, biofilm formation, cell surface hydrophobicity, and antifungal planktonic susceptibility: relationship among Candida spp
Source: Front Microbiol. 2015 Mar 12;6:205. doi: 10.3389/fmicb.2015.00205 (PMC4357307; doi:10.3389/fmicb.2015.00205)
Supplement: Supplementary file 1 [file Table1.DOCX]

**Table S1- Characterization of *Candida* adhesion profile, biofilm formation and hydrophobicity.** For each *Candida* strain the percentage of cells with adherent polystyrene microspheres was quantified and the homogenic or heterogenic adhesion pattern was attributed. Based in these attributes an adhesion profile was established. Biofilm formation at 24h and 48h was evaluated by two distinct methodologies, as well as cell surface hydrophobicity. Each result represents the mean of at least 3 independent experiments performed in triplicate.

| Species | Strain | Adhesion | | | Biofilm | | | | | Hydrophobicity |
| --- | --- | --- | --- | --- | --- | --- | --- | --- | --- | --- |
|  |  | **% of cells with adherent microspheres** | **Distribution Pattern** | **Adhesion Profile** | | **XTT (24H)** | **XTT (48H)** | **CV (24H)** | **CV (48H)** |  |
| *C. albicans* | 12 | 2,80 | Homogenic | Low | | 0,19 | 0,20 | 2,08 | 1,46 | ND |
| *C. albicans* | 28 | 1,97 | Homogenic | Low | | 0,18 | 0,18 | 1,23 | 1,51 | 21,02 |
| *C. albicans* | 63 | 3,27 | Homogenic | Low | | 0,19 | 0,19 | 2,10 | 1,37 | ND |
| *C. albicans* | 69 | 2,74 | Homogenic | Low | | 0,22 | 0,25 | 1,88 | 1,83 | ND |
| *C. albicans* | 90 | 3,15 | Homogenic | Low | | 0,24 | 0,28 | 1,97 | 2,30 | ND |
| *C. albicans* | 93 | 3,10 | Homogenic | Low | | 0,25 | 0,33 | 2,12 | 3,73 | ND |
| *C. albicans* | 129 | 2,05 | Homogenic | Low | | 0,20 | 0,22 | 0,80 | 2,33 | ND |
| *C. albicans* | 143 | 1,64 | Homogenic | Low | | 0,24 | 0,29 | 1,75 | 5,21 | 3,51 |
| *C. albicans* | 175 | 2,40 | Homogenic | Low | | 0,39 | 0,33 | 0,32 | 0,04 | ND |
| *C. albicans* | O16 | 2,26 | Homogenic | Low | | 0,35 | 0,40 | 2,84 | 2,73 | 3,29 |
| *C. albicans* | O19 | 2,93 | Homogenic | Low | | 0,37 | 0,35 | 0,27 | 0,42 | ND |
| *C. albicans* | O32 | 2,79 | Homogenic | Low | | 0,37 | 0,33 | 0,02 | 0,12 | ND |
| *C. albicans* | O47 | 2,80 | Homogenic | Low | | 0,37 | 0,34 | 2,97 | 1,90 | ND |
| *C. albicans* | O63 | 4,98 | Homogenic | Low | | 0,38 | 0,40 | 5,77 | 5,23 | ND |
| *C. albicans* | O143 | 2,02 | Homogenic | Low | | 0,26 | 0,24 | 0,43 | 1,66 | ND |
| *C. albicans* | O176 | 2,15 | Homogenic | Low | | 0,22 | 0,24 | 0,25 | 1,86 | ND |
| *C. albicans* | O178 | 1,99 | Homogenic | Low | | 0,22 | 0,27 | 0,18 | 2,50 | 16,27 |
| *C. albicans* | OL002 | 2,53 | Homogenic | Low | | 0,09 | 0,26 | 0,47 | 0,53 | 11,38 |
| *C. albicans* | OL008 | 2,05 | Homogenic | Low | | 0,30 | 0,31 | 0,73 | 1,02 | ND |
| *C. albicans* | OL009 | 1,84 | Homogenic | Low | | 0,36 | 0,38 | 0,93 | 1,15 | ND |
| *C. albicans* | OL010 | 1,74 | Homogenic | Low | | 0,35 | 0,38 | 1,49 | 2,13 | ND |
| *C. albicans* | OL011 | 1,88 | Homogenic | Low | | 0,30 | 0,30 | 1,55 | 1,75 | ND |
| *C. albicans* | OL015 | 1,90 | Homogenic | Low | | 0,36 | 0,36 | 4,01 | 3,37 | ND |
| *C. albicans* | OL019 | 2,18 | Homogenic | Low | | 0,35 | 0,34 | 0,07 | 0,02 | ND |
| *C. albicans* | OL020 | 2,16 | Homogenic | Low | | 0,36 | 0,35 | 0,04 | 0,02 | ND |
| *C. albicans* | OL040 | 2,27 | Homogenic | Low | | 0,36 | 0,34 | 0,24 | 0,76 | ND |
| *C. albicans* | OL060 | 6,39 | Homogenic | Low | | 0,38 | 0,36 | 4,23 | 3,19 | 19,26 |
| *C. albicans* | OL063 | 3,78 | Homogenic | Low | | 0,38 | 0,35 | 2,98 | 2,30 | ND |
| *C. albicans* | OL102 | 7,64 | Homogenic | Low | | 0,37 | 0,37 | 4,08 | 3,01 | 25,06 |
| *C. albicans* | OL136 | 2,06 | Homogenic | Low | | 0,26 | 0,37 | 0,99 | 1,41 | ND |
| *C. albicans* | OL028 | 1,42 | Homogenic | Low | | 0,21 | 0,22 | 2,76 | 4,93 | 9,06 |
| *C. albicans* | OL035 | 1,54 | Homogenic | Low | | 0,22 | 0,24 | 2,71 | 2,37 | 29,05 |
| *C. albicans* | OL075 | 2,00 | Homogenic | Low | | 0,25 | 0,30 | 2,99 | 2,87 | ND |
| *C. albicans* | OL018 | 1,73 | Homogenic | Low | | 0,14 | 0,32 | 0,27 | 0,47 | ND |
| *C. albicans* | OL084 | 1,82 | Homogenic | Low | | 0,20 | 0,24 | 2,53 | 2,24 | ND |
| *C. albicans* | OL085 | 1,72 | Homogenic | Low | | 0,25 | 0,33 | 3,29 | 3,02 | ND |
| *C. albicans* | OL145 | 2,07 | Homogenic | Low | | 0,35 | 0,34 | 0,01 | 0,04 | ND |
| *C. albicans* | OL146 | 2,82 | Homogenic | Low | | 0,35 | 0,34 | 0,05 | 0,04 | ND |
| *C. albicans* | OL003 | 6,22 | Homogenic | Low | | 0,30 | 0,37 | 1,10 | 1,06 | ND |
| *C. albicans* | OL046 | 1,78 | Homogenic | Low | | 0,36 | 0,39 | 1,53 | 1,59 | ND |
| *C. albicans* | OL057 | 3,20 | Homogenic | Low | | 0,36 | 0,35 | 3,04 | 0,48 | 0 |
| *C. albicans* | OL068 | 1,83 | Homogenic | Low | | 0,35 | 0,34 | 0,25 | 0,04 | ND |
| *C. albicans* | OL168 | 8,92 | Homogenic | Low | | 0,12 | 0,32 | 0,59 | 1,24 | 11,83 |
| *C. albicans* | OL014 | 1,77 | Homogenic | Low | | 0,24 | 0,24 | 1,03 | 0,79 | ND |
| *C. albicans* | OL001 | 1,89 | Homogenic | Low | | 0,38 | 0,41 | 4,66 | 6,58 | ND |
| *C. albicans* | MC416 | 5,44 | Homogenic | Low | | 0,36 | 0,37 | 3,15 | 2,97 | 0 |
| *C. albicans* | MC437 | 4,40 | Homogenic | Low | | 0,35 | 0,35 | 1,28 | 1,74 | 0 |
| *C. albicans* | MC439 | 3,87 | Homogenic | Low | | 0,39 | 0,39 | 2,63 | 2,86 | 0 |
| *C. albicans* | MC440 | 4,12 | Homogenic | Low | | 0,39 | 0,39 | 2,32 | 3,90 | 0 |
| *C. albicans* | ATCC 90028 | 2,76 | Homogenic | Low | | 0,31 | 0,32 | 2,19 | 1,90 | 0 |
| *C.glabrata* | 42 | 7,49 | Homogenic | Low | | 0,16 | 0,30 | 1,92 | 1,65 | ND |
| *C.glabrata* | 43 | 4,53 | Homogenic | Low | | 0,15 | 0,29 | 2,82 | 1,31 | ND |
| *C.glabrata* | 54 | 6,41 | Homogenic | Low | | 0,19 | 0,32 | 3,12 | 1,77 | ND |
| *C.glabrata* | 78 | 3,28 | Homogenic | Low | | 0,33 | 0,38 | 6,33 | 5,36 | ND |
| *C.glabrata* | 80 | 8,01 | Homogenic | Low | | 0,33 | 0,37 | 7,9975 | 8,35 | ND |
| *C.glabrata* | 85 | 9,33 | Homogenic | Low | | 0,43 | 0,46 | 8,74 | 4,12 | ND |
| *C.glabrata* | 96 | 18,94 | Heterogenic | Intermediate | | 0,29 | 0,35 | 2,19 | 2,16 | 24,38 |
| *C.glabrata* | 113 | 1,60 | Homogenic | Low | | 0,28 | 0,33 | 1,49 | 0,95 | 6,28 |
| *C.glabrata* | 121 | 1,11 | Homogenic | Low | | 0,33 | 0,40 | 1,26 | 0,86 | 10,01 |
| *C.glabrata* | OO1 | 8,90 | Homogenic | Low | | 0,35 | 0,36 | 1,36 | 0,96 | ND |
| *C.glabrata* | OO4 | 9,12 | Homogenic | Low | | 0,33 | 0,37 | 0,62 | 0,73 | ND |
| *C.glabrata* | O12 | 16,10 | Heterogenic | Intermediate | | 0,34 | 0,37 | 0,78 | 0,52 | 11,80 |
| *C.glabrata* | O13 | 8,93 | Homogenic | Low | | 0,36 | 0,41 | 0,32 | 2,10 | ND |
| *C.glabrata* | O40 | 4,21 | Homogenic | Low | | 0,36 | 0,39 | 2,22 | 1,31 | ND |
| *C.glabrata* | O92 | 1,66 | Homogenic | Low | | 0,32 | 0,37 | 0,90 | 1,23 | ND |
| *C.glabrata* | O155 | 2,35 | Homogenic | Low | | 0,17 | 0,33 | 2,20 | 1,72 | ND |
| *C.glabrata* | O177 | 2,21 | Homogenic | Low | | 0,13 | 0,29 | 0,18 | 0,64 | ND |
| *C.glabrata* | O179 | 3,02 | Homogenic | Low | | 0,18 | 0,31 | 0,08 | 0,46 | ND |
| *C.glabrata* | O180 | 1,61 | Homogenic | Low | | 0,14 | 0,29 | 0,56 | 0,23 | ND |
| *C.glabrata* | OL013 | 2,94 | Homogenic | Low | | 0,21 | 0,31 | 0,08 | 0,58 | ND |
| *C.glabrata* | OL150 | 2,69 | Homogenic | Low | | 0,36 | 0,34 | 1,49 | 0,08 | ND |
| *C.glabrata* | OL151 | 1,50 | Homogenic | Low | | 0,17 | 0,29 | 0,30 | 0,46 | ND |
| *C.glabrata* | OL157 | 1,67 | Homogenic | Low | | 0,17 | 0,26 | 0,05 | 0,33 | ND |
| *C.glabrata* | OL149 | 2,02 | Homogenic | Low | | 0,17 | 0,29 | 0,50 | 0,28 | ND |
| *C.glabrata* | OL154 | 1,42 | Homogenic | Low | | 0,14 | 0,25 | 0,06 | 1,45 | 23,15 |
| *C.glabrata* | OL125 | 5,17 | Homogenic | Low | | 0,37 | 0,34 | 1,21 | 1,01 | ND |
| *C.glabrata* | OL039 | 2,62 | Homogenic | Low | | 0,22 | 0,31 | 0,27 | 1,03 | ND |
| *C.glabrata* | OL147 | 1,78 | Homogenic | Low | | 0,18 | 0,30 | 0,40 | 0,62 | 0 |
| *C.glabrata* | OL148 | 2,13 | Homogenic | Low | | 0,16 | 0,30 | 0,16 | 0,41 | ND |
| *C.glabrata* | OL152 | 1,84 | Homogenic | Low | | 0,14 | 0,29 | 0,33 | 0,29 | ND |
| *C.glabrata* | OL153 | 3,87 | Homogenic | Low | | 0,41 | 0,35 | 1,94 | 1,13 | ND |
| *C.glabrata* | OL155 | 1,49 | Homogenic | Low | | 0,18 | 0,28 | 0,47 | 0,44 | ND |
| *C.glabrata* | OL058 | 1,82 | Homogenic | Low | | 0,17 | 0,26 | 0,47 | 0,40 | 14,81 |
| *C.glabrata* | OL069 | 2,17 | Homogenic | Low | | 0,21 | 0,31 | 1,07 | 1,46 | ND |
| *C.glabrata* | OL044 | 6,50 | Homogenic | Low | | 0,23 | 0,31 | 2,04 | 2,48 | ND |
| *C.glabrata* | OL045 | 1,73 | Homogenic | Low | | 0,16 | 0,27 | 1,98 | 1,81 | ND |
| *C.glabrata* | OL048 | 4,70 | Homogenic | Low | | 0,17 | 0,23 | 7,48 | 8,74 | ND |
| *C.glabrata* | OL071 | 13,81 | Heterogenic | Intermediate | | 0,19 | 0,22 | 4,25 | 6,63 | 28,54 |
| *C.glabrata* | OL074 | 13,17 | Heterogenic | Intermediate | | 0,38 | 0,35 | 1,14 | 0,02 | 0 |
| *C.glabrata* | OL156 | 1,63 | Homogenic | Low | | 0,20 | 0,29 | 0,02 | 0,28 | ND |
| *C.glabrata* | OL158 | 1,82 | Homogenic | Low | | 0,17 | 0,26 | 0,47 | 0,40 | ND |
| *C.glabrata* | OL042 | 3,81 | Homogenic | Low | | 0,27 | 0,31 | 4,43 | 5,88 | ND |
| *C.glabrata* | OL090 | 9,95 | Homogenic | Low | | 0,12 | 0,24 | 2,93 | 4,48 | 43,53 |
| *C.glabrata* | OL098 | 1,70 | Homogenic | Low | | 0,17 | 0,30 | 0,32 | 0,55 | 0 |
| *C.glabrata* | MC425 | 5,22 | Homogenic | Low | | 0,38 | 0,43 | 2,49 | 3,15 | 29,297 |
| *C.glabrata* | MC426 | 4,23 | Homogenic | Low | | 0,38 | 0,39 | 1,62 | 1,33 | 0 |
| *C.glabrata* | MC369 | 2,06 | Homogenic | Low | | 0,374 | 0,36 | 1,50 | 0,03 | 5,37 |
| *C.glabrata* | MC370 | 2,33 | Homogenic | Low | | 0,37 | 0,36 | 1,00 | 0,06 | 0 |
| *C. parapsilosis* | 4 | 35,35 | Heterogenic | High | | 0,21 | 0,24 | 2,73 | 4,80 | ND |
| *C. parapsilosis* | 11 | 24,39 | Heterogenic | Intermediate | | 0,05 | 0,02 | 2,26 | 3,86 | ND |
| *C. parapsilosis* | 37 | 5,56 | Homogenic | Low | | 0,08 | 0,09 | 0,24 | 0,48 | ND |
| *C. parapsilosis* | 24 | 3,06 | Homogenic | Low | | 0,077 | 0,049 | 0,55 | 0,42 | ND |
| *C. parapsilosis* | 58 | 4,745 | Homogenic | Low | | 0,053 | 0,10 | 0,64 | 0,34 | ND |
| *C. parapsilosis* | 61 | 2,66 | Homogenic | Low | | 0,03 | 0,07 | 0,24 | 0,65 | ND |
| *C. parapsilosis* | 91 | 19,77 | Heterogenic | Intermediate | | 0,26 | 0,24 | 5,11 | 10,34 | ND |
| *C. parapsilosis* | 97 | 19,34 | Heterogenic | Intermediate | | 0,21 | 0,18 | 8,89 | 10,41 | ND |
| *C. parapsilosis* | 108 | 33,56 | Heterogenic | Low | | 0,17 | 0,13 | 5,19 | 8,21 | ND |
| *C. parapsilosis* | 109 | 14,42 | Heterogenic | Intermediate | | 0,12 | 0,09 | 4,52 | 6,53 | ND |
| *C. parapsilosis* | O21 | 31,60 | Heterogenic | High | | 0,05 | 0,12 | 7,61 | 8,25 | ND |
| *C. parapsilosis* | O24 | 8,81 | Homogenic | Low | | 0,23 | 0,26 | 1,38 | 2,39 | ND |
| *C. parapsilosis* | O39 | 2,31 | Homogenic | Low | | 0,39 | 0,34 | 1,46 | 1,62 | ND |
| *C. parapsilosis* | O122 | 2,52 | Homogenic | Low | | 0,03 | 0,09 | 0,00 | 0,45 | 9,27 |
| *C. parapsilosis* | O170 | 2,11 | Homogenic | Low | | 0,07 | 0,11 | 0,09 | 0,34 | 0 |
| *C. parapsilosis* | O174 | 2,25 | Homogenic | Low | | 0,06 | 0,10 | 1,04 | 1,72 | ND |
| *C. parapsilosis* | OL007 | 18,26 | Heterogenic | Intermediate | | 0,17 | 0,11 | 8,16 | 8,13 | 46,14 |
| *C. parapsilosis* | OL049 | 2,91 | Homogenic | Low | | 0,18 | 0,16 | 0,46 | 0,80 | ND |
| *C. parapsilosis* | OL059 | 31,6 | Heterogenic | High | | 0,06 | 0,11 | 10,02 | 8,93 | 51,77 |
| *C. parapsilosis* | OL021 | 30,93 | Heterogenic | High | | 0,05 | 0,15 | 8,39 | 8,87 | 19,00 |
| *C. parapsilosis* | OL033 | 21,78 | Heterogenic | Intermediate | | 0,04 | 0,04 | 0,27 | 0,67 | ND |
| *C. parapsilosis* | OL056 | 10,05 | Homogenic | Intermediate | | 0,06 | 0,21 | 1,25 | 0,17 | ND |
| *C. parapsilosis* | OL066 | 7,96 | Homogenic | Low | | 0,05 | 0,05 | 0,73 | 0,97 | ND |
| *C. parapsilosis* | OL135 | 22,85 | Heterogenic | Intermediate | | 0,18 | 0,14 | 3,70 | 3,46 | ND |
| *C. parapsilosis* | OL144 | 2,89 | Homogenic | Low | | 0,04 | 0,09 | 0,00 | 0,225 | 5,76 |
| *C. parapsilosis* | OL030 | 13,89 | Heterogenic | Intermediate | | 0,05 | 0,07 | 0,59 | 0,95 | ND |
| *C. parapsilosis* | OL095 | 33,05 | Heterogenic | High | | 0,06 | 0,13 | 5,92 | 9,01 | ND |
| *C. parapsilosis* | OL032 | 13,56 | Heterogenic | Intermediate | | 0,05 | 0,07 | 0,31 | 0,83 | ND |
| *C. parapsilosis* | OL043 | 14,04 | Heterogenic | Intermediate | | 0,21 | 0,26 | 6,23 | 7,06 | ND |
| *C. parapsilosis* | OL054 | 2,08 | Homogenic | Low | | 0,05 | 0,17 | 0,98 | 0,13 | ND |
| *C. parapsilosis* | OL055 | 25,27 | Heterogenic | High | | 0,02 | 0,10 | 9,07 | 9,22 | ND |
| *C. parapsilosis* | OL094 | 30,97 | Heterogenic | High | | 0,05 | 0,10 | 5,32 | 5,91 | 20,34 |
| *C. parapsilosis* | OL096 | 2,43 | Homogenic | Low | | 0,02 | 0,14 | 0,87 | 0,11 | 35,30 |
| *C. parapsilosis* | OL116 | 9,10 | Homogenic | Low | | 0,18 | 0,15 | 3,30 | 3,23 | ND |
| *C. parapsilosis* | OL031 | 4,68 | Homogenic | Low | | 0,03 | 0,06 | 0,00 | 0,66 | ND |
| *C. parapsilosis* | OL051 | 3,52 | Homogenic | Low | | 0,07 | 0,10 | 0,28 | 0,74 | ND |
| *C. parapsilosis* | OL073 | 5,51 | Homogenic | Low | | 0,03 | 0,07 | 0,50 | 0,09 | ND |
| *C. parapsilosis* | OL089 | 4,75 | Homogenic | Low | | 0,25 | 0,30 | 1,70 | 2,13 | ND |
| *C. parapsilosis* | OL065 | 51,05 | Heterogenic | High | | 0,04 | 0,13 | 8,74 | 8,77 | 37,21 |
| *C. parapsilosis* | OL121 | 8,80 | Homogenic | Low | | 0,16 | 0,15 | 1,92 | 4,11 | 19,00 |
| *C. parapsilosis* | OL036 | 1,78 | Homogenic | Low | | 0,08 | 0,08 | 0,00 | 0,65 | 26,74 |
| *C. parapsilosis* | OL143 | 4,91 | Homogenic | Low | | 0,07 | 0,03 | 1,44 | 4,86 | ND |
| *C. parapsilosis* | OL067 | 4,08 | Homogenic | Low | | 0,16 | 0,26 | 1,11 | 0,26 | ND |
| *C. parapsilosis* | MC405 | 39,59 | Heterogenic | High | | 0,30 | 0,18 | 5,41 | 6,49 | 52,18 |
| *C. parapsilosis* | MC409 | 29,59 | Heterogenic | High | | 0,33 | 0,20 | 5,99 | 7,33 | 10,69 |
| *C. parapsilosis* | MC428 | 41,99 | Heterogenic | High | | 0,23 | 0,12 | 5,48 | 6,50 | 26,32 |
| *C. parapsilosis* | MC429 | 41,23 | Heterogenic | High | | 0,23 | 0,11 | 5,11 | 6,60 | 46,53 |
| *C.tropicalis* | 35 | 22,97 | Heterogenic | Intermediate | | 0,08 | 0,14 | 5,99 | 6,12 | ND |
| *C.tropicalis* | 41 | 23,37 | Heterogenic | Intermediate | | 0,33 | 0,36 | 8,95 | 10,74 | ND |
| *C.tropicalis* | 51 | 34,18 | Heterogenic | High | | 0,37 | 0,40 | 7,67 | 8,85 | ND |
| *C.tropicalis* | 76 | 5,62 | Homogenic | Low | | 0,05 | 0,07 | 5,26 | 5,02 | 68,17 |
| *C.tropicalis* | 105 | 58,01 | Heterogenic | High | | 0,36 | 0,37 | 6,01 | 7,60 | 19,35 |
| *C.tropicalis* | 122 | 14,79 | Heterogenic | Intermediate | | 0,36 | 0,37 | 6,52 | 6,80 | ND |
| *C.tropicalis* | 152 | 3,20 | Homogenic | Low | | 0,39 | 0,40 | 5,74 | 5,62 | 49,68 |
| *C.tropicalis* | 170 | 12,18 | Heterogenic | Intermediate | | 0,37 | 0,36 | 4,20 | 4,81 | ND |
| *C.tropicalis* | 176 | 8,02 | Homogenic | Low | | 0,36 | 0,48 | 7,88 | 11,13 | 6,14 |
| *C.tropicalis* | OO6 | 28,74 | Heterogenic | High | | 0,35 | 0,40 | 7,75 | 11,01 | 57,00 |
| *C.tropicalis* | O61 | 5,47 | Homogenic | Low | | 0,36 | 0,40 | 7,63 | 9,15 | ND |
| *C.tropicalis* | O77 | 9,17 | Homogenic | Low | | 0,34 | 0,41 | 5,79 | 6,24 | 53,07 |
| *C.tropicalis* | O152 | 18,05 | Heterogenic | Intermediate | | 0,35 | 0,38 | 9,21 | 11,96 | ND |
| *C.tropicalis* | O164 | 2,94 | Homogenic | Low | | 0,36 | 0,39 | 3,80 | 3,74 | ND |
| *C.tropicalis* | O167 | 34,53 | Heterogenic | High | | 0,04 | 0,10 | 7,47 | 9,03 | 7,75 |
| *C.tropicalis* | OL017 | 35,91 | Heterogenic | High | | 0,36 | 0,38 | 8,38 | 10,02 | 59,26 |
| *C.tropicalis* | OL038 | 31,58 | Heterogenic | High | | 0,03 | 0,10 | 5,54 | 6,73 | 37,38 |
| *C.tropicalis* | OL053 | 24,20 | Heterogenic | Intermediate | | 0,03 | 0,036 | 6,23 | 5,63 | 37,71 |
| *C.tropicalis* | OL141 | 8,01 | Homogenic | Low | | 0,04 | 0,074 | 7,27 | 5,21 | ND |
| *C.tropicalis* | OL006 | 16,50 | Heterogenic | Intermediate | | 0,01 | 0,06 | 7,24 | 7,28 | 35,75 |
| *C.tropicalis* | MC418 | 58,70 | Heterogenic | High | | 0,36 | 0,347 | 6,33 | 7,82 | 49,39 |
| *C.tropicalis* | MC374 | 51,47 | Heterogenic | High | | 0,38 | 0,36 | 7,34 | 8,37 | 43,87 |
| *C.tropicalis* | MC375 | 51,08 | Heterogenic | High | | 0,37 | 0,33 | 7,18 | 8,76 | 49,03 |
| *C.tropicalis* | MC407 | 5,945 | Homogenic | Low | | 0,37 | 0,38 | 6,88 | 7,80 | 59,88 |
| *C.krusei* | O14 | 11,61 | Homogenic | Intermediate | |  |  |  |  |  |
| *C.krusei* | O131 | 11,61 | Homogenic | Intermediate | | 0,27 | 0,40 | 4,54 | 5,79 | ND |
| *C.krusei* | OL012 | 10,63 | Homogenic | Intermediate | | 0,29 | 0,34 | 3,27 | 4,70 | ND |
| *C.krusei* | OL091 | 4,25 | Homogenic | Low | | 0,13 | 0,16 | 2,38 | 6,10 | ND |
| *C.krusei* | OL101 | 6,57 | Homogenic | Low | | 0,25 | 0,36 | 3,12 | 5,14 | ND |
| *C.krusei* | OL099 | 9,98 | Homogenic | Low | | 0,32 | 0,40 | 2,31 | 5,12 | ND |
| *C.krusei* | OL103 | 12,57 | Homogenic | Intermediate | | 0,37 | 0,38 | 6,13 | 7,18 | ND |
| *C.krusei* | OL109 | 3,61 | Homogenic | Low | | 0,31 | 0,35 | 4,67 | 4,75 | ND |
| *C. guilliermondii* | 32 | 45,35 | Heterogenic | High | | 0,36 | 0,39 | 7,93 | 4,60 | ND |
| *C. guilliermondii* | 33 | 43,70 | Heterogenic | High | | 0,35 | 0,38 | 9,13 | 5,66 | ND |
| *C. guilliermondii* | OL072 | 20,31 | Heterogenic | Intermediate | | 0,22 | 0,34 | 7,31 | 3,17 | ND |
| *C. guilliermondii* | OL077 | 15,13 | Heterogenic | Intermediate | | 0,42 | 0,43 | 2,87 | 4,13 | ND |
| *C. guilliermondii* | MC23 | 39,13 | Heterogenic | High | | 0,17 | 0,33 | 7,73 | 3,33 | ND |
| *C. guilliermondii* | MC27 | 50,13 | Heterogenic | High | | 0,45 | 0,48 | 8,31 | 4,14 | ND |
| *C. guilliermondii* | MC37 | 10,32 | Homogenic | Intermediate | | 0,44 | 0,47 | 4,85 | 7,31 | ND |
| *C. guilliermondii* | MC38 | 35,12 | Heterogenic | High | | 0,37 | 0,39 | 8,33 | 3,95 | ND |
