# Supplementary material for: Adhesion, biofilm formation, cell surface hydrophobicity, and antifungal planktonic susceptibility: relationship among Candida spp
Source: Front Microbiol. 2015 Mar 12;6:205. doi: 10.3389/fmicb.2015.00205 (PMC4357307; doi:10.3389/fmicb.2015.00205)
Supplement: Supplementary file 2 [file Table2.DOCX]

**Table S2-** **Classification of adhesion profiles.** New adhesion profiles were created based in the percentage of cells with adherent microspheres values and the distribution pattern found for the tested strains.

| Adhesion Profile | Percentage of cells with adherent microspheres | Distribution Pattern |
| --- | --- | --- |
| Low Adhesion Profile | 1-10 | Homogenic |
| Intermediate Adhesion Profile | 10-15 | Homogenic |
|  | 10-30 | Heterogenic |
| High Adhesion Profile | ≥30 | Heterogenic |
