# Supplementary material for: Adhesion, biofilm formation, cell surface hydrophobicity, and antifungal planktonic susceptibility: relationship among Candida spp
Source: Front Microbiol. 2015 Mar 12;6:205. doi: 10.3389/fmicb.2015.00205 (PMC4357307; doi:10.3389/fmicb.2015.00205)
Supplement: Supplementary file 3 [file Table3.DOCX]

**Table S3-** *Candida* planktonic susceptibility. *Candida* susceptibility was evaluated to fluconazole (FLC), amphotericin B (AMB) and caspofungine (CAS) accordingly to the CLSI standard protocol M27-A3 S4. Strains were classified in S (susceptible), R (resistant), SDD (susceptible-dose-dependent) and I (intermediate) based in available breakpoints.

| Isolate | Strain | Antifungal Susceptibility  (MIC/Phenotype) | | |  |
| --- | --- | --- | --- | --- | --- |
|  |  | FLC | AMB | CAS | |
| *C. albicans* | 12 | >64/R | 0.5/S | 0.125/S | |
| *C. albicans* | 28 | >64/R | 0.5/S | 0.06/S | |
| *C. albicans* | 63 | >64/R | 0.25/S | 0.06/S | |
| *C. albicans* | 69 | >64/R | 0.5/S | 0.5/I | |
| *C. albicans* | 90 | >64/R | 0.5/S | 0.25/S | |
| *C. albicans* | 93 | >64/R | 0.25/S | 4/R | |
| *C. albicans* | 129 | 4/SDD | 0.25/S | 1/R | |
| *C. albicans* | 143 | 2/S | 0.25/S | 0.25/S | |
| *C. albicans* | 175 | 2/S | 0.25/S | 1/R | |
| *C. albicans* | O16 | 0.25/S | 0.25/S | 0.06/S | |
| *C. albicans* | O19 | 0.5/S | ≤0.06/S | 0.06/ | |
| *C. albicans* | O32 | 0.25/S | 0.25/S | 0.06/S | |
| *C. albicans* | O47 | 0.25/S | 0.5/S | 0.06/S | |
| *C. albicans* | O63 | 0.25/S | 0.25/S | 0.06/S | |
| *C. albicans* | O143 | 0.125/S | 0.125/S | ≤0.06/S | |
| *C. albicans* | O176 | 0.25/S | 0.5/S | 0.06/S | |
| *C. albicans* | O178 | 0.25/S | 0.5/S | 0.06/S | |
| *C. albicans* | OL002 | 0.25/S | 0.125/S | ≤0.06/S | |
| *C. albicans* | OL008 | 0.25/S | 0.25/S | ≤0.06/S | |
| *C. albicans* | OL009 | 0.25/S | 0.125/S | ≤0.06/S | |
| *C. albicans* | OL010 | 0.25/S | 0.125/S | ≤0.06/S | |
| *C. albicans* | OL011 | 0.25/S | ≤0.06/S | ≤0.06/S | |
| *C. albicans* | OL015 | 1/S | ≤0.06/S | ≤0.06/S | |
| *C. albicans* | OL019 | ≤0.125/S | 0.125/S | ≤0.06/S | |
| *C. albicans* | OL020 | 0.25/S | 0.125/S | 0.06/S | |
| *C. albicans* | OL040 | 0.25/S | 0.5/S | ≤0.06/S | |
| *C. albicans* | OL060 | 0.25/S | 0.25/S | ≤0.06/S | |
| *C. albicans* | OL063 | ≤0.125/S | 0.25/S | ≤0.06/S | |
| *C. albicans* | OL102 | 0.25/S | 0.25/S | ≤0.06/S | |
| *C. albicans* | OL136 | 0.5/S | 0.5/S | ≤0.06/S | |
| *C. albicans* | OL028 | 0.25/S | 1/S | ≤0.06/S | |
| *C. albicans* | OL035 | 0.25/S | 0.5/S | 0.06/S | |
| *C. albicans* | OL075 | 0.25/S | 0.25/S | ≤0.06/S | |
| *C. albicans* | OL018 | 0.25/S | ≤0.06/S | ≤0.06/S | |
| *C. albicans* | OL084 | 0.25/S | 0.5/S | ≤0.06/S | |
| *C. albicans* | OL085 | 0.25/S | 0.5/S | ≤0.06/S | |
| *C. albicans* | OL145 | ≤0.125/S | ≤0.06/S | ≤0.06/S | |
| *C. albicans* | OL146 | 0.25/S | 0.25/S | ≤0.06/S | |
| *C. albicans* | OL003 | 0.5/S | 0.125/S | ≤0.06/S | |
| *C. albicans* | OL046 | 0.25/S | 0.25/S | ≤0.06/S | |
| *C. albicans* | OL057 | 0.25/S | 0.25/S | ≤0.06/S | |
| *C. albicans* | OL068 | 0.25/S | 0.25/S | ≤0.06/S | |
| *C. albicans* | OL168 | 0.25/S | 0.25/S | 0.06/S | |
| *C. albicans* | OL014 | 0.5/S | 0.25/S | 0.125/S | |
| *C. albicans* | OL001 | 1/S | 0.25/S | ≤0.06/S | |
| *C. albicans* | MC416 | <0.125/S | 1/S | 0.25/S | |
| *C. albicans* | MC437 | 0.5/S | 0.06/S | 0.06/S | |
| *C. albicans* | MC439 | 1/S | 0.06/S | 0.125/S | |
| *C. albicans* | MC440 | 0.25/S | 0.06/S | ≤0.03/S | |
| *C. albicans* | ATCC90028 | 0.5/S | 0.25/S | 0.25/S | |
| *C. glabrata* | 42 | 16/SDD | 0.25/S | 0.125/S | |
| *C. glabrata* | 43 | 4/SDD | 0.5/S | 0.06/S | |
| *C. glabrata* | 54 | 8/SDD | 0.5/S | 0.06/S | |
| *C. glabrata* | 78 | 8/SDD | 0.25/S | 1/R | |
| *C. glabrata* | 80 | 8/SDD | 0.25/S | 1/R | |
| *C. glabrata* | 85 | 2/SDD | 2/R | 1/R | |
| *C. glabrata* | 96 | 2/SDD | 0.125/S | 8/R | |
| *C. glabrata* | 113 | 2/SDD | 0.25/S | 1/R | |
| *C. glabrata* | 121 | 8/SDD | 0.25/S | 1/R | |
| *C. glabrata* | OO1 | 32/SDD | 0.25/S | 0.25/I | |
| *C. glabrata* | OO4 | 16/SDD | 0.25/S | 0.25/I | |
| *C. glabrata* | O12 | 16/SDD | 0.25/S | 0.06/S | |
| *C. glabrata* | O13 | >64/R | 0.25/S | 0.25/I | |
| *C. glabrata* | O40 | 8/SDD | 0.25/S | 0.125/S | |
| *C. glabrata* | O92 | 2/SDD | 0.25/S | 0.125/S | |
| *C. glabrata* | O155 | 32/SDD | 0.25/S | 0.125/S | |
| *C. glabrata* | O177 | 16/SDD | 0.25/S | 0.125/S | |
| *C. glabrata* | O179 | 4/SDD | 0.25/S | 0.125/S | |
| *C. glabrata* | O180 | 1/SDD | 0.25/S | 0.125/S | |
| *C. glabrata* | OL013 | 16/SDD | 0.25/S | 0.125/S | |
| *C. glabrata* | OL150 | 1/SDD | 0.25/S | 0.125/S | |
| *C. glabrata* | OL151 | 8/SDD | 0.5/S | 0.06/S | |
| *C. glabrata* | OL157 | 8/SDD | 0.5/S | 0.125/S | |
| *C. glabrata* | OL149 | 1/SDD | 0.5/S | ≤0.06/S | |
| *C. glabrata* | OL154 | 16/SDD | 0.125/S | 0.125/S | |
| *C. glabrata* | OL125 | 32/SDD | 0.5/S | 0.25/S | |
| *C. glabrata* | OL039 | 8/SDD | 0.5/S | 0.125/S | |
| *C. glabrata* | OL147 | 8/SDD | 0.5/S | 0.06/S | |
| *C. glabrata* | OL148 | 16/SDD | 0.125/S | 0.125/S | |
| *C. glabrata* | OL152 | 2/SDD | 0.125/S | 0.125/S | |
| *C. glabrata* | OL153 | 1/SDD | 0.125/S | 0.25/I | |
| *C. glabrata* | OL155 | 8/SDD | 0.25/S | 0.06/S | |
| *C. glabrata* | OL058 | 8/SDD | 0.25/S | 0.125/S | |
| *C. glabrata* | OL069 | 8/SDD | 0.5/S | 0.125/S | |
| *C. glabrata* | OL044 | 1/SDD | 0.5/S | 0.25/I | |
| *C. glabrata* | OL045 | 4/SDD | 0.5/S | 0.25/I | |
| *C. glabrata* | OL048 | 1/SDD | 0.25/S | 0.125/S | |
| *C. glabrata* | OL071 | 8/SDD | 0.25/S | 0.25/I | |
| *C. glabrata* | OL074 | 8/SDD | 0.25/S | ≤0.06/S | |
| *C. glabrata* | OL156 | 8/SDD | 0.5/S | 0.06/S | |
| *C. glabrata* | OL158 | 8/SDD | 0.5/S | 32/R | |
| *C. glabrata* | OL042 | 1/SDD | 0.25/S | 1/R | |
| *C. glabrata* | OL090 | 8/SDD | 0.5/S | 0.125/S | |
| *C. glabrata* | OL098 | 16/SDD | 0.5/S | 0.125/S | |
| *C. glabrata* | MC425 | 32/SDD | 0.25/S | <0.06/S | |
| *C. glabrata* | MC426 | 32/SDD | 0.25/S | <0.06/S | |
| *C. glabrata* | MC369 | 16/SDD | 0.25/S | <0.06/S | |
| *C. glabrata* | MC370 | 16/SDD | 0.25/S | <0.06/S | |
| *C. parapsilosis* | 4 | 2/S | 0.5/S | 4/I | |
| *C. parapsilosis* | 11 | 1/S | 0.5/S | 4/I | |
| *C. parapsilosis* | 37 | 0.5/S | 0.125/S | >32/R | |
| *C. parapsilosis* | 24 | 2/S | 0.25/S | 4/I | |
| *C. parapsilosis* | 58 | 1/S | 0.25/S | 4/I | |
| *C. parapsilosis* | 61 | >64/R | 0.5/S | 2/S | |
| *C. parapsilosis* | 91 | 1/S | 0.06/S | 16/R | |
| *C. parapsilosis* | 97 | 0.5/S | 0.5/S | 4/I | |
| *C. parapsilosis* | 108 | 1/S | 0.25/S | 4/I | |
| *C. parapsilosis* | 109 | 2/S | 0.25/S | 4/I | |
| *C. parapsilosis* | O21 | 1/S | 0.25/S | 4/I | |
| *C. parapsilosis* | O24 | 1/S | 0.5/S | 8/R | |
| *C. parapsilosis* | O39 | 2/S | 0.5/S | 0.25/S | |
| *C. parapsilosis* | O122 | 1/S | 0.25/S | 0.5/S | |
| *C. parapsilosis* | O170 | 2/S | 0.25/S | 0.25/S | |
| *C. parapsilosis* | O174 | 2/S | 0.25/S | 0.5/S | |
| *C. parapsilosis* | OL007 | 0.25/S | 0.5/S | 1/S | |
| *C. parapsilosis* | OL049 | 2/S | 0.125/S | 4/I | |
| *C. parapsilosis* | OL059 | 1/S | 1/S | 2/S | |
| *C. parapsilosis* | OL021 | 0.5/S | 4/R | 1/S | |
| *C. parapsilosis* | OL033 | 1/S | 0.25/S | 4/I | |
| *C. parapsilosis* | OL056 | 0.5/S | 0.5/S | 2/S | |
| *C. parapsilosis* | OL066 | 2/S | 0.5/S | 1/S | |
| *C. parapsilosis* | OL135 | 2/S | 0.5/S | 1/S | |
| *C. parapsilosis* | OL144 | 4/I | 0.25/S | 1/S | |
| *C. parapsilosis* | OL030 | 1/S | 0.25/S | 4/I | |
| *C. parapsilosis* | OL095 | 0.5/S | 1/S | 4/I | |
| *C. parapsilosis* | OL032 | 0.5/S | 1/S | 0.5/S | |
| *C. parapsilosis* | OL043 | 2/S | 0.5/S | 0.5/S | |
| *C. parapsilosis* | OL054 | 0.5/S | 0.25/S | 0.5/S | |
| *C. parapsilosis* | OL055 | 0.5/S | 0.25/S | 0.5/S | |
| *C. parapsilosis* | OL094 | 0.25/S | 1/S | 2/S | |
| *C. parapsilosis* | OL096 | 0.25/S | 1/S | 1/S | |
| *C. parapsilosis* | OL116 | 1/S | 0.125/S | 0.5/S | |
| *C. parapsilosis* | OL031 | 0.5/S | 0.25/S | 4/I | |
| *C. parapsilosis* | OL051 | 0.25/S | 0.5/S | 4/I | |
| *C. parapsilosis* | OL073 | 2/S | 0.5/S | 2/S | |
| *C. parapsilosis* | OL089 | 0.25/S | 0.5/S | 2/S | |
| *C. parapsilosis* | OL065 | 0.5/S | 0.25/S | 0.5/S | |
| *C. parapsilosis* | OL121 | 1/S | 0.5/S | 1/S | |
| *C. parapsilosis* | OL036 | 16/R | 0.25/S | 8/R | |
| *C. parapsilosis* | OL143 | 0.5/S | 0.25/S | 0.5/S | |
| *C. parapsilosis* | OL067 | 1/S | 1/S | 1/S | |
| *C. parapsilosis* | MC405 | 0.5/S | 0.125/S | 0.5/S | |
| *C. parapsilosis* | MC409 | 0.5/S | 0.5/S | 0.5/S | |
| *C. parapsilosis* | MC428 | 2/S | 0.25/S | 0.5/S | |
| *C. parapsilosis* | MC429 | 2/S | 0.5/S | 0.25/S | |
| *C. tropicalis* | 35 | >64/R | 0.5/S | 0.25/S | |
| *C. tropicalis* | 41 | 16/R | 0.5/S | 0.06/S | |
| *C. tropicalis* | 51 | >64/R | 0.5/S | 0.5/I | |
| *C. tropicalis* | 76 | 1/S | 0.5/S | 1/R | |
| *C. tropicalis* | 105 | 2/S | 1/S | 0.5/I | |
| *C. tropicalis* | 122 | 1/S | 0.25/S | 0.5/I | |
| *C. tropicalis* | 152 | >64/R | 1/S | 1/R | |
| *C. tropicalis* | 170 | >64/R | 0.5/S | 1/R | |
| *C. tropicalis* | 176 | >64/R | 0.5/S | 32/R | |
| *C. tropicalis* | OO6 | 4/SDD | ≤0.06/S | 0.06/S | |
| *C. tropicalis* | O61 | 0.25/S | 0.125/S | ≤0.06/S | |
| *C. tropicalis* | O77 | 0.5/S | 0.25/S | ≤0.06/S | |
| *C. tropicalis* | O152 | 0.5/S | 0.25/S | ≤0.06/S | |
| *C. tropicalis* | O164 | 0.5/S | 0.125/S | ≤0.06/S | |
| *C. tropicalis* | O167 | ≤0.125/S | 0.5/S | ≤0.06/S | |
| *C. tropicalis* | OL017 | 0.5/S | 0.25/S | ≤0.06/S | |
| *C. tropicalis* | OL038 | 0.5/S | 0.25/S | 0.06/S | |
| *C. tropicalis* | OL053 | 0.5/S | 0.25/S | 0.06/S | |
| *C. tropicalis* | OL141 | 1/S | 0.25/S | 0.06/S | |
| *C. tropicalis* | OL006 | 0.5/S | 0.25/S | ≤0.06/S | |
| *C. tropicalis* | MC418 | 0.25/S | 1/S | 0.125/S | |
| *C. tropicalis* | MC374 | 1/S | 0.25/S | <0.06/S | |
| *C. tropicalis* | MC375 | 1/S | 0.5/S | <0.06/S | |
| *C. tropicalis* | MC407 | 2/S | 1/S | 0.5/I | |
| *C. krusei* | O14 | 64/R | 0.5/S | 1/R | |
| *C. krusei* | O131 | 64/R | 0.5/S | 0.25/S | |
| *C. krusei* | OL012 | 64/R | 0.5/S | 0.5/I | |
| *C. krusei* | OL091 | >64/R | 1/S | 0.25/S | |
| *C. krusei* | OL101 | >64/R | 0.5/S | 0.5/I | |
| *C. krusei* | OL099 | >64/R | 1/S | 0.5/I | |
| *C. krusei* | OL103 | 64/R | 0.5/S | 0.5/I | |
| *C. krusei* | OL109 | >64/R | 0.5/S | 0.5/I | |
| *C. guilliermondii* | 32 | 4/S | 0.5/S | 4/I | |
| *C. guilliermondii* | 33 | 2/S | 0.5/S | >32/R | |
| *C. guilliermondii* | OL072 | 4/S | 0.5/S | 8/R | |
| *C. guilliermondii* | OL077 | 4/S | 0.5/S | 8/R | |
| *C. guilliermondii* | MC23 | 8/S | 0.125/S | >32/R | |
| *C. guilliermondii* | MC27 | 16/SDD | 1/S | >32/R | |
| *C. guilliermondii* | MC37 | 8/S | 0.125/S | <0.06/S | |
| *C. guilliermondii* | MC38 | 4/S | 0.25/S | 8/R | |
