# Supplementary figures and images for: Adhesion, biofilm formation, cell surface hydrophobicity, and antifungal planktonic susceptibility: relationship among Candida spp
Source: Front Microbiol. 2015 Mar 12;6:205. doi: 10.3389/fmicb.2015.00205 (PMC4357307; doi:10.3389/fmicb.2015.00205)

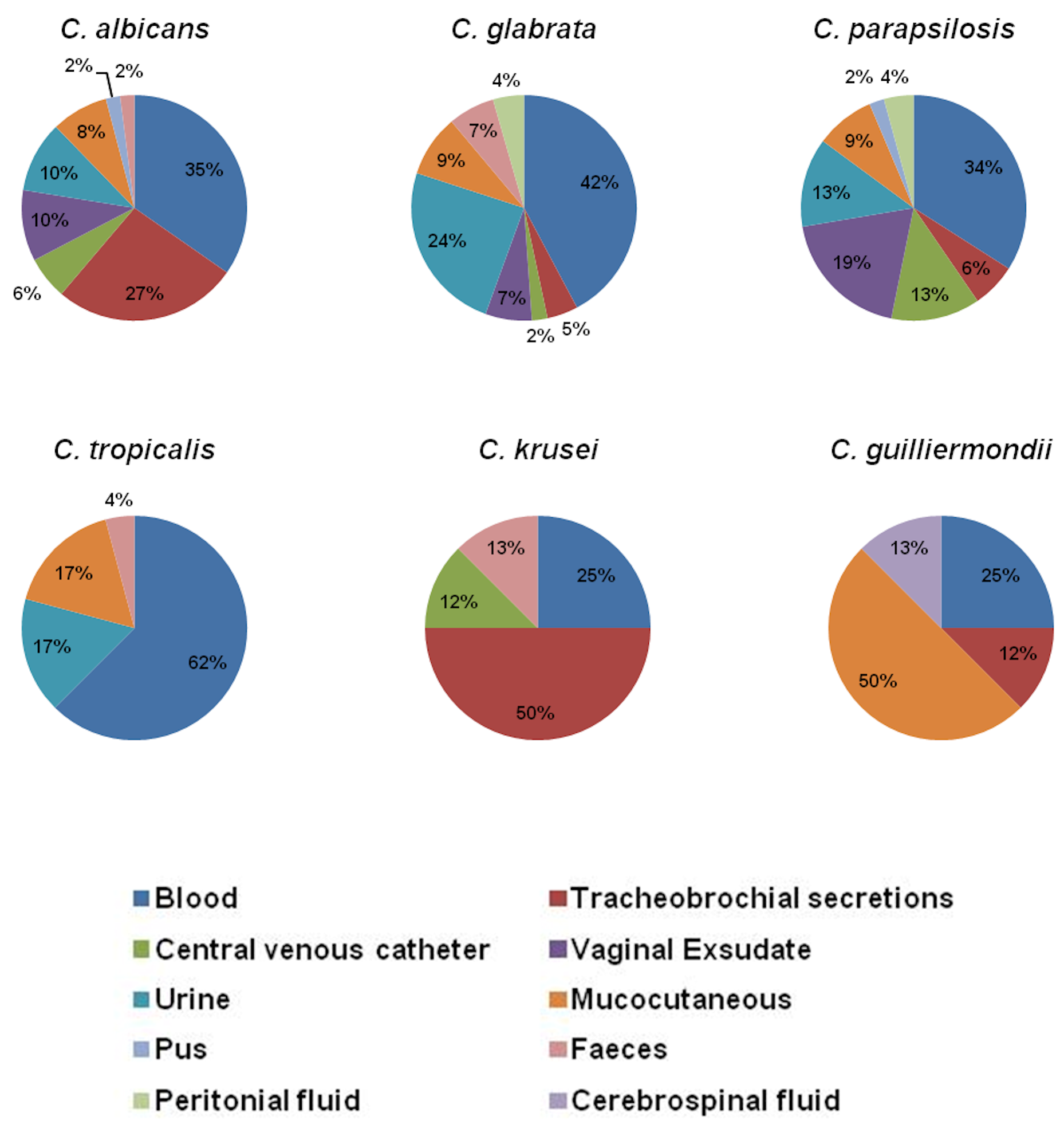

Supplement: Supplementary file 4 [file Image1.TIF]
